# Supplementary material for: Insights on 21 Years of HBV Surveillance in Blood Donors in France
Source: Viruses. 2022 Nov 12;14(11):2507. doi: 10.3390/v14112507 (PMC9693332; doi:10.3390/v14112507)
Supplement: Supplementary file 1 [file viruses-14-02507-s001.zip › viruses-2000776-supplementary.pdf]

viruses

MDPI

Article

Insights on 21 years of HBV surveillance in blood donors in France

Pierre CAPPY[a,b*], Laure BOIZEAU[a,b*], Daniel CANDOTTI[a,b], Sophie LE CAM[c], Christophe MARTINAUD[d], Josiane PILLONEL[e], Martin TRIBOUT[f], Claude MAUGARD[f], Josiane RELAVE[f], Pascale RICHARD[c], Pascal MOREL[c,g] and Syria LAPERCHE[b,c]

a) Department of Microbiology, Henri Mondor University Hospital, Assistance Publique-Hôpitaux de Paris, Créteil, France

b) National Reference Centre for Transfusion Infectious Risks, Institut National de la Transfusion Sanguine (INTS), Paris, France

c) Etablissement Français du Sang (EFS), La Plaine St-Denis, France

d) Centre de transfusion des Armées (CTSA), Clamart, France

e) Santé Publique France, Saint Maurice, France

f) Etablissement Français du Sang Occitanie (EFS Occitanie), France

g) UMR 1098 RIGHT, INSERM, Université de Franche-Comté, Besançon, France

* Both authors contributed equally to this paper.

* Correspondence: pierre.cappy@aphp.fr

SUPPLEMENTARY MATERIAL

Supplementary Table S1. Number and rates of donations and donors screened for HBV from 2000 to 2020 in France.

**Donation year**	**2000**	**2001**	**2002**	**2003**	**2004**	**2005**	**2006**	**2007**	**2008**	**2009**	**2010**
Number of donations	2 475 102	2 409 484	2 459 663	2 465 382	2 498 298	2 512 795	2 575 273	2 713 582	2 818 689	3 001 321	3 004 863
Number of donors	1 530 000	1 522 000	1 537 000	1 523 000	1 535 900	1 506 082	1 527 209	1 635 578	1 643 493	1 701 018	1 662 994
Number of FTBD	386 000	382 000	352 000	358 733	382 640	352 348	363 175	395 919	416 367	435 693	368 076
Number of RBD	1 144 000	1 140 000	1 185 000	1 164 267	1 153 260	1 153 734	1 164 034	1 239 659	1 227 126	1 265 325	1 294 918
HBV all donors	438	444	433	451	424	351	333	381	344	327	234
Rate per 10 000 donations	1,77	1,84	1,76	1,83	1,70	1,40	1,29	1,40	1,22	1,09	0.78
HBV in FTBD	431	434	424	447	420	346	327	375	339	322	224
Prevalence in FTBD (per 10 000)	11,2	11,4	12,0	12,5	11,0	9,8	9,0	9,5	8,1	7,4	6,1
Incidence[1] (per 100 000 PY)			1.66			0.4			0.98		
(CI95%)			(1.2-2.29)			(0.2-0.78)			(0.64-1.48)		

**Donation year**	**2011**	**2012**	**2013**	**2014**	**2015**	**2016**	**2017**	**2018**	**2019**	**2020**	**Total**
Number of donations	3 149 090	3 038 143	2 760 422	2 813 170	2 958 120	2 918 611	2 958 128	2 926 942	2 891 828	2 812 078	**58 160 984**
Number of donors	1 718 320	1 705 209	1 639 942	1 600 072	1 660 599	1 616 268	1 624 221	1 636 524	1 622 519	1 589 491	**33 737 439**
Number of FTBD	397 010	382 955	357 510	350 333	325 813	279 454	288 969	293 456	289 966	256 262	**7 414 679**
Number of RBD	1 321 310	1 322 254	1 282 432	1 249 739	1 334 786	1 336 814	1 335 252	1 343 068	1 332 553	1 333 229	**26 322 760**
HBV all donors	264	280	223	256	225	165	189	139	143	105	**6 149**
Rate per 10 000 donations	0,84	0,92	0,81	0,91	0,76	0,57	0,64	0,47	0,49	0,37	**1,06**
HBV in FTBD	257	272	220	255	217	162	178	137	138	100	**6 025**
Prevalence in FTBD (per 10 000)	6,5	7,1	6,2	7,3	6,7	5,8	6,2	4,7	4,8	3,9	**8,13**
Incidence (per 100 000 PY)	0.61			0.26			0.74			0.26	
(CI95%)	(0.37-1.02)			(0.11-0.56)			(0.46-1.17)			(0.11-0.57)	

FTBD: first time blood donor, RBD: Repeat blood donor, PY: persons/years
[1]: estimated in repeat blood donors in 3 year-periods
